# Model Team in the ICU: does the implementation of intensive care assistants affect ICU nursing activity?

**DOI:** 10.1186/cc9890

**Published:** 2011-03-11

**Authors:** KB Tang

**Affiliations:** 1 North District Hospital, Hong Kong

## Introduction

The healthcare workforce shortage is a global phenomenon, especially in the ICU. Use of a register nurse-unlicenced assistive personal model is an undeniable reality that fills the void created. Model Team is a structured training program for healthcare assistants to expand their role, facilitating them to perform nursing tasks that require nursing skill and knowledge. The purpose of the study is to investigate whether the Model Team approach could reduce bedside nursing activities.

## Methods

This was a prospective cohort study. All bedside nurses working in an ICU were recruited. Intensive care assistants have undergone 3-month structuralized training for specific nursing skills, and then served four ICU beds under the supervision of a bedside nurse. Activities of all involved nurses were recorded before and after the implementation of an intensive care assistant service using the work-sampling method. Activities were categorized into six groups: patient care activities consigned to TISS-28; patient care activities not indicated in TISS-28; patient care activities that are not interventions in direct contact with the patient; organizational activities; personal activities; and miscellaneous activities [1]. The TISS-28 score of each patient was recorded during both sampling periods, serving as an indicator for complexity of nursing activity. A statistical test was performed to compare the frequency of patient care activities related to TISS-28 score (Question A) and nursing activities not related to direct patient contact (Questions C, D, E and F), before and after the Modal Team approach.

## Results

In total 29 nurses were recruited, 14 nurses during the control period and 15 nurses after the Model Team approach. Patients in both periods were comparable with no significant difference in TISS-28 score. Patient care activities related to TISS-28 score reduced by 16.33% (mean frequency 3.43 to 2.87, *P *= 0.249) after the implementation of the intensive care assistant, but were not statistically significant. For nursing activities not related to bedside care, there was an insignificant increase of 1.67% (mean frequency 4.79 to 4.87, *P *= 0.448).

## Conclusions

The Model Team approach may reduce bedside nursing activities, without effect on nonbedside nursing activities. Further study with a larger sample size should be done to test the hypothesis.
